# Crosstalk between Heme Oxygenase-1 and Iron Metabolism in Macrophages: Implications for the Modulation of Inflammation and Immunity

**DOI:** 10.3390/antiox11050861

**Published:** 2022-04-27

**Authors:** Joseana de Oliveira, Marina B. Denadai, Diego L. Costa

**Affiliations:** 1 Departamento de Bioquímica e Imunologia, Faculdade de Medicina de Ribeirão Preto, Universidade de São Paulo, Ribeirao Preto 14049-900, Brazil; de_oliveira@usp.br (J.d.O.); marina.bd@usp.br (M.B.D.); 2 Programa de Pós-Graduação em Imunologia Básica e Aplicada, Faculdade de Medicina de Ribeirão Preto, Universidade de São Paulo, Ribeirao Preto 14049-900, Brazil

**Keywords:** heme oxygenase-1, iron, macrophages, immunity, inflammation

## Abstract

Heme oxygenase-1 (HO-1) is an enzyme that catalyzes the degradation of heme, releasing equimolar amounts of carbon monoxide (CO), biliverdin (BV), and iron. The anti-inflammatory and antioxidant properties of HO-1 activity are conferred in part by the release of CO and BV and are extensively characterized. However, iron constitutes an important product of HO-1 activity involved in the regulation of several cellular biological processes. The macrophage-mediated recycling of heme molecules, in particular those contained in hemoglobin, constitutes the major mechanism through which living organisms acquire iron. This process is finely regulated by the activities of HO-1 and of the iron exporter protein ferroportin. The expression of both proteins can be induced or suppressed in response to pro- and anti-inflammatory stimuli in macrophages from different tissues, which alters the intracellular iron concentrations of these cells. As we discuss in this review article, changes in intracellular iron levels play important roles in the regulation of cellular oxidation reactions as well as in the transcriptional and translational regulation of the expression of proteins related to inflammation and immune responses, and therefore, iron metabolism represents a potential target for the development of novel therapeutic strategies focused on the modulation of immunity and inflammation.

## 1. Introduction

Heme oxygenase-1 (HO-1) is an enzyme encoded by the Hmox1 gene and its main function is to degrade heme molecules into three sub products: carbon monoxide gas (CO), iron (Fe^2+^), and biliverdin; the latter is converted into bilirubin by the action of biliverdin reductase [1,2]. Due to its activity in heme metabolism, HO-1 is constitutively expressed in macrophages from tissues involved in the recycling of erythrocytes and hemoglobin, such as bone marrow (BM), spleen, and liver [3]. In addition, HO-1 expression can be induced in response to a variety of stress signals in different cell populations, but specially in macrophages from different tissues of the organism [4].

Heme, the substrate of HO-1, is a tetrapyrrolic cofactor of extreme importance for living organisms due to its role as a major oxygen (O_2_) transporter. Composed of a protoporphyrin IX ring complexed to an iron ion, heme participates in several functions in the body, such as cellular respiration, electron transport, modulation of reactive oxygen species, as well as regulation of transcription and gene translation [5,6]. Heme is synthesized in the mitochondria and cytosol of developing erythroid progenitor cells and is further conjugated to hemoglobin molecules, which are abundantly present in mature erythrocytes [7]. However, this molecule is also commonly found in macrophages that perform the physiological process of recycling senescent red blood cells [8].

The intracellular accumulation of heme is harmful to the organism and triggers cellular and tissue damage due to its highly pro-oxidant nature. Genotoxicity, induction of paraptosis in endothelial cells, and consequent dysfunction in the angiogenesis process are examples of detrimental effects caused by heme accumulation in different cells and tissues [9,10]. HO-1 is widely known for its antioxidant properties and, in this sense, the catalysis of heme degradation by the enzyme activity alone can be considered as an important antioxidant function of HO-1 [11,12,13,14]. In humans, HO-1 deficiency induces high sensitivity to oxidative stress, intravascular hemolysis, perturbations of iron homeostasis, kidney, liver, and endothelial inflammation [15,16,17].

Particularly in macrophages, HO-1 and its products play important roles in the regulation of inflammatory and immune responses. In fact, HO-1 along with the products CO and biliverdin/bilirubin are classically associated with the promotion of antioxidant, anti-inflammatory, and immunosuppressive activities in macrophages [4]. A classic example was demonstrated by Lee and Chau, who found that the inhibition of the enzyme activity blocks the anti-inflammatory activities of IL-10 in murine macrophages stimulated with LPS, resulting in increased production of TNF [18]. Because of that, HO-1 induction has been proposed as a therapeutic strategy to treat several inflammatory conditions, such as autoimmune diseases, while its inhibition has been suggested as an approach to promote improved immunity and resistance to many infectious diseases caused by intracellular pathogens [4,19]. The exception to this rule applies to viral infections, in which HO-1 has been shown to play majorly host-protective roles. These effects are associated to reduction of inflammation and oxidative stress-driven cellular and tissue damage as well as to roles of heme degradation products in blocking the assembly of viral particles in infected cells [20]. However, the iron ions released following HO-1-mediated heme degradation display opposing pro-oxidant effects and can also play important immunomodulatory effects [21,22].

In the following sections, we will review general aspects of heme metabolization by HO-1 in macrophages with special focus on the crosstalk of HO-1 activity and iron metabolism as well as the effects on the regulation of inflammation and immune responses.

## 2. Heme Acquisition by Macrophages

### 2.1. Erythrophagocytosis

**Figure 1:**
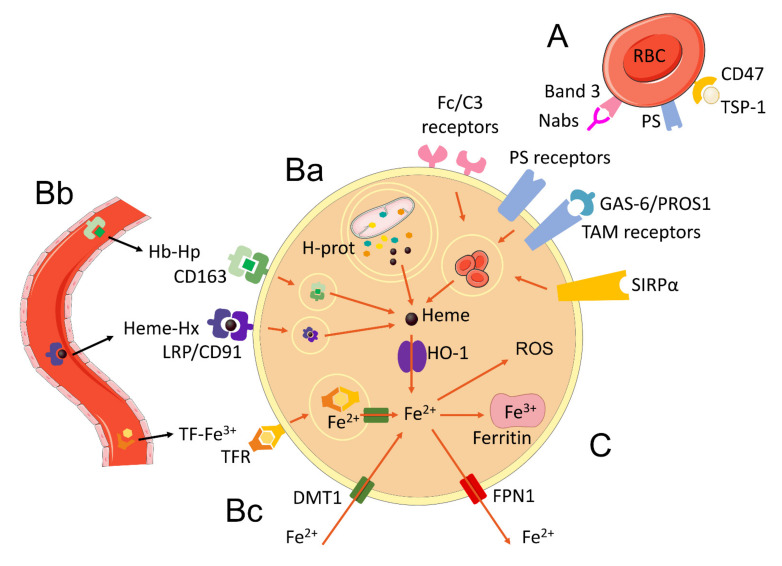
Major mechanisms of iron acquisition by macrophages. (**A**) Phagocytosis and recycling of senescent erythrocytes by macrophages occur through the recognition of Nabs (natural antibodies) bound to Band3 antigens and complement by Fc and C3 receptors, recognition of phosphatidylserine (PS) by PS receptors and TAM receptors bridged by GAS6 and PROS1 ligands or recognition of CD47 bound by TSP-1 by SIRPα receptors. (**Ba**) Autophagy of hemoproteins (H-prot) found free in the cytoplasm or present in mitochondria (mitophagy). (**Bb**) Endocytosis of circulating extracellular hemoglobin (Hb)-haptoglobin (Hp) and heme-hemopexin complexes through CD163 and LPR/CD91 receptors respectively. These mechanisms result in the release of heme molecules in the cytosol, which are metabolized by HO-1 and release Fe^2+^ iron. Extracellular circulating transferrin (TF)-Fe^3+^ complexes are recognized by transferrin receptors (TFR) and endocytosed, releasing ferric iron that is converted to ferrous (Fe^2+^) iron and transported to the cytosol through the DMT1 receptor. (**Bc**) DMT1 receptor can also internalize extracellular free Fe^2+^ ions. (**C**) Intracellular free iron can be stored in ferritin molecules in the ferric (Fe^3+^) form or transported to the extracellular compartment through ferroportin. Free intracellular ferrous iron promotes the generation of reactive oxygen species through the Fenton reaction. Some elements in this figure use pictures from Servier Medical Art (https://smart.servier.com, last accessed on 30 March 2022) licensed under a Creative Commons Attribution 3.0 Unported License (https://creativecommons.org/licenses/by/3.0/, last accessed on 30 March 2022).

Erythrocytes (red blood cells—RBCs) promote the transport of O_2_ for cellular respiration. The average life span of these cells is approximately 120 days, after which they undergo structural changes and enter senescence [23]. Senescent RBCs express molecules in their membranes known as “eat me” signals, which will be recognized by receptors expressed by macrophages, in particular those from the splenic red pulp and liver (Kupffer cells—KCs) [24,25]. Some of these signals include: (a) The formation of Band 3 (RBC surface protein) clusters in senescent RBC membrane, which are bound by naturally occurring antibodies (Nabs) and activate complement, being further recognized by Fc or C3 receptors in macrophages [25,26,27]; (b) exposure of phosphatidylserine (PS) in the extracellular portion of the membrane, which can be directly bound by PS receptors in macrophages, such as Tim-1, Tim,4, CD300, and Stabilin-2, or can bind to GAS-6 or PROS1 that will be further recognized by TAM receptors in macrophages [25,28,29,30,31,32,33]; (c) expression of CD47 on the RBC surface and its interaction with thrombospondin-1 (TSP-1), which bind to the signal-regulatory receptor protein alpha (SIRPα) present in macrophages [34,35]. All of those signals trigger the phagocytic machinery of macrophages that result in phagocytosis of senescent RBC (Figure 1A).

The human organism acquires the vast majority of its iron through the process of erythrophagocytosis and recycling of senescent RBCs. Approximately 1 billion iron atoms are extracted from almost 280 million of hemoglobin molecules per erythrocyte [36]. Adult humans have about 25 trillion RBCs, and each second, we recycle about 5 million of them by erythrophagocytosis in macrophages of the reticuloendothelial system [37,38].

### 2.2. Haptoglobin and Hemopexin

Hemolytic processes result in the release of hemoglobin and heme into the bloodstream. Free hemoglobin and heme can promote the development of several pathological processes through their pro-oxidant nature as well as their pro-inflammatory and prothrombotic activity [39,40,41,42,43,44,45,46,47,48,49,50]. However, it was shown that hemopexin overexpression in the liver of model mice for sickle cell anemia was able to inhibit the inflammatory activity and vascular stasis caused by heme [51].

Haptoglobin (Hp) and hemopexin (Hx) are plasma glycoproteins produced mainly by hepatic cells, which retain high binding affinity with free hemoglobin and heme, respectively [52,53,54]. Hp (alpha—2 glycoprotein) is composed of an alpha and a beta chain with approximately 328–388 amino acids each [55,56], while Hx has 439 amino acid residues divided into two homologous domains, N-domain and C-domain, in which both have four-bladed β-propeller fold helix [53,54].

Free hemoglobin in plasma can undergo structural changes caused by H_2_O_2_ that interfere with its internalization by cells, which causes its accumulation in the blood. The formation of the hemoglobin–Hp complex prevents H_2_O_2_ from modifying amino acids in the beta-globulin chain, thus limiting cross-link reactions that would occur with the alpha-globulin chain [57]. The Hb–Hp complex is recognized by the CD163 transporter in the surface of monocytes and macrophages and is further endocytosed by these cells. Following CD163-mediated internalization, hemoglobin–Hp complexes will be degraded in the lysosome, resulting in the release of heme molecules [55,58] (Figure 1Bb). Although Hp has been identified as a critical mediator in the clearance of free hemoglobin from the plasma, CD163 has also been shown to be able to directly internalize free hemoglobin not bound to Hp [59]. At the end of this process, CD163 is recycled and returns to the cell surface [55,58].

Hx stably binds free heme at a pH greater than 5.0 and undergoes a conformational change that prevents additional binding of peptides to its structure, consequently protecting the complex from proteolysis [53,54]. The Hx–heme complexes are recognized by the low-density lipoprotein receptor-related protein (LRP)/CD91, which are present on the surface of macrophages but also in several other cell types, such as fibroblasts, hepatocytes, neurons, adipocytes, syncytiotrophoblasts, and columnar epithelial cells of the gastrointestinal tract [60]. Following LRP/CD91-mediated internalization of Hx–heme complexes by endocytosis, Hx is degraded by lysosomal enzymes releasing the heme molecules (Figure 1Bb) and the LRP/CD91 receptor is recycled to the cell surface [61].

### 2.3. Autophagy of Hemoproteins and Mitophagy

Hemoproteins/heme proteins comprise a group of more than 2300 proteins that have one or more heme groups in their structure [62,63]. They can be classified into different types and contain heme complexed to the amino acids in different forms as well, such as heme a, heme b, heme c, heme or heme o. Hemoproteins perform different functions within the cells, which range from transport, storage, and activation of O_2_ molecules; electron transfer; and substrate for oxidation reactions [64,65]. Therefore, hemoproteins are normally found in cell cytoplasm and mitochondria. Among the more than two thousand hemoproteins present in the databases, we can mention myoglobin, cytochrome P450, cytochrome c, catalase, and iNOS, which were extensively characterized [65,66,67].

In addition to the various forms of heme acquisition presented in the previous sections, the cell is also able to obtain heme via its synthesis in the inner membrane of the mitochondrial matrix. Once its production occurs, heme is routed to be incorporated among the hemoproteins present in the mitochondria and cellular cytoplasm [68]. Thus, it is implied that the process of autophagy or mitophagy caused by cellular or mitochondrial damage, inflammatory stimuli, or regular processes of organelle recycling by the cells [69], can also cause the release of free heme within the cell (Figure 1Ba).

## 3. Heme Degradation by HO-1 and Iron Release

After heme is released from hemoglobin, Hx–heme complexes or from other hemoproteins in phagolysosomes or autophagolysosomes, and is transferred to the cytosol by the heme transporters heme-carrier protein 1 (HCP1) and heme responsive gene 1 protein (HRG1). Following their transport to the cytoplasm, heme molecules are then metabolized by HO-1, which is anchored to the membranes of the endoplasmic reticulum [8,24,37,70,71]. As mentioned previously, HO-1 is constitutively expressed in macrophages involved in the recycling of RBCs in the liver and the spleen [8]. However, the enzyme expression can also be induced in different cells in the organism in response to several cellular stressors, such as ultraviolet radiation, endotoxins, heavy metals, physical stress, heme-containing enzymes, and ROS [72]. The major signaling pathway responsible for the induction of HO-1 expression involves the action of the nuclear transcription factor erythroid 2p45-related factor 2 (Nrf2) [4]. Nrf2 is normally found in the cytosol in its inactive form, bound to the protein Kelch-like ECH-associated protein 1 (Keap1), which promotes the ubiquitination of Nrf2 and consequent proteasomal degradation of the transcription factor. However, under oxidative stress, Keap1 undergoes oxidation of its cysteine residues and releases Nrf2, which migrates to the nucleus and binds, in conjunction with small Maf proteins, to stress-responsive DNA sequence elements (StREs) containing Maf recognition element sequences (MARE). StREs/MARE are present upstream of the HO-1 gene, and therefore, binding of Nrf2 to these regions, results in induction of enzyme expression [72,73,74,75,76]. In homeostatic conditions, the MARE sequences in HO-1 gene promoter are found complexed to the transcription repressor Bach1, which prevents the induction of HO-1 expression. However, concurrently to Nrf2 activation, the accumulation of free heme favors the binding of these molecules to Bach1, which results in the release of this repressor from the MARE regions, therefore promoting expression of HO-1 [77,78,79].

As previously introduced, heme cleavage by HO-1 results in the release of equimolar amounts of CO, biliverdin, and iron as end products of the enzymatic reaction [8]. CO and biliverdin, which is further converted into bilirubin by the enzyme biliverdin reductase, display important antioxidant and anti-inflammatory functions within the cell and account for much of the antioxidant and anti-inflammatory effects of HO-1 activity itself. However, those will not be discussed in depth in the present review article, but have been extensively reviewed elsewhere [4,80,81,82,83,84].

Iron, the third product of heme degradation by HO-1, is an essential ion for the organism. Free Fe2^+^ in the cytoplasm, also known as labile iron, is involved in several vital processes in the cell, such as cellular respiration, oxygen sensing and metabolism, cell signaling, energy metabolism, as well as DNA synthesis and repair. However, free Fe^2+^ is highly reactive and can promote the production of ROS by the Fenton reaction, which can consequently cause oxidative damage to cellular components [8,85]. Because of that, the cell prevents the cytotoxic effects of iron by promoting the conversion of Fe^2+^ ions to the ferric Fe^3+^ form, which is further stored intracellularly, or by exporting the Fe^2+^ to the extracellular environment. Iron storage occurs through a multimeric protein complex called ferritin (FT), which is composed of heavy (H—ferritin heavy/heart chain—FTH) and light (L—ferritin light/liver chain—FTL) chains [86,87]. FTH is responsible for catalyzing Fe^2+^ into Fe^3+^ by ferroxidase, forming ferrihydrite aggregates, which are inert and incapable of generating free radicals. FTH chains provide stability to the ferritin structure but also assist in the formation of inorganic ferrihydrite aggregates. It is estimated that one ferritin molecule can store as much as 4500 iron atoms [87]. Alternatively, if Fe^2+^ is not used by the cell or stored in ferritin molecules, this ion is directed to be exported out of the cell through the transmembrane transporter ferroportin (FPN1), encoded by the gene SLC40A1 (Solute Carrier Family 40 Member 1) (Figure 1C) [88,89].

Macrophages can also acquire iron through other ways besides heme metabolization by HO-1. In the serum, iron is oxidized by ceruplasmin and majorly converted to the Fe^3+^ form, which will then be complexed to transferrin (each transferrin molecule can accommodate two iron ions) [90]. Iron-loaded transferrin is recognized by the transferrin receptor (TFR) on the surface of macrophages and endocytosed. Inside the endosomal compartment, Fe^3+^ is reduced to ferrous iron (Fe^2+^) by the six-transmembrane epithelial antigen of prostate (STEAP3) enzyme and further transported into the cytosol through the divalent metal transporter 1 (DMT1), which is a transmembrane glycoprotein that can only transport iron in its ferrous (Fe^2+^) form (Figure 1Bb). Following this, TFR is subsequently recycled back to the cell surface [91,92,93]. DMT1 is also commonly found in the plasma membrane, where it promotes the internalization of extracellular free iron ions [94]. In the cell surface, ferric iron is reduced to its ferrous form by cytochrome B DCYTB and subsequently internalized though DMT1 (Figure 1Bc) [86]. Macrophages can further mobilize iron by nuclear receptor coactivator 4 (NCOA4)-induced autophagy of iron loaded ferritin molecules (ferritinophagy). Ferritin is then degraded and iron ions are transported to the cytosol through the same mechanisms described earlier [92].

HO-1 and FPN1 expression in macrophages play a pivotal role in the systemic iron homeostasis. The genetic deletion of HO-1 profoundly affects iron levels in the body, causing anemia and iron accumulation inside cells in several tissues [95]. The deficiency of ferroportin gene in macrophages was also shown to result in the development of anemia and iron accumulation in the spleen, liver, and BM [96]. Ferroportin expression can be regulated transcriptionally or through a post-translational mechanism by the action of hepcidin, a peptide hormone secreted by liver cells in response to increases in serum iron concentration or inflammation [97]. Hepcidin binds to ferroportin in the surface of cells and induces its internalization and further degradation. As a consequence, the export of iron ions to the extracellular environment ceases and the metal accumulates inside the cells. Accordingly, overproduction of hepcidin leads to tissue iron overload and hypoferremia [98].

## 4. Cross Regulation of Iron Homeostasis, Inflammation, and Immunity

As mentioned previously, serum iron levels can regulate the production of hepcidin and consequently, the expression of ferroportin in cell membranes. High serum iron levels induce the production of hepcidin, which promotes the degradation of ferroportin and ceases further export of iron to serum, while in situations of low iron levels, hepcidin expression is suppressed, favoring ferroportin expression and promoting iron export to the circulation [97]. However, hepcidin production can also be induced in macrophages in response to inflammatory stimuli, the most studied of which is IL-6 biding to its receptor and subsequent activation of signal transducer and activator of transcription 3 (STAT3) signaling pathway [24].

Hepcidin itself has antibacterial properties, however, its main role in the immune response to infectious diseases has been associated to the induction of nutritional immunity or “hypoferremia of inflammation” [99,100]. The production of hepcidin by macrophages and other cells in response to infection-derived stimuli is intended to decrease ferroportin expression and consequently limit iron bioavailability to pathogens [24]. Armitage et al. demonstrated that pathogen-derived Toll-like receptor 5 agonists stimulate hepcidin production by leukocytes and hepatoma cells in an IL-6-dependent manner, while IL-22, an important cytokine produced in response to extracellular infections, also induces phosphorylation of STAT3 and upregulation of hepcidin production. The authors additionally found that following in vivo infection with *C. albicans* or Influenza A/PR/8/34 virus (H1N1), hepcidin expression is upregulated causing a decrease in serum iron levels in mice [101]. Intraperitoneal challenge with *Pseudomonas aeruginosa* was also shown to induce TLR4-dependent hepcidin expression and consequent iron deposition in splenic macrophages [102]. More recently, Abreu et al. demonstrated that activation of TLRs 1/2 or TLR 2/6 induces a decrease in ferroportin expression by direct inhibition of gene transcription, independently of hepcidin, while activation of TLRs 4, 7, and 8 induces hepcidin expression and further ferroportin downregulation [103].

The studies cited above illustrate how the hepcidin/ferroportin axis can be modulated in response to infectious stimuli as well as cytokines produced in response to those triggers, consequently affecting systemic and local iron levels. However, some of the pro-inflammatory signals that trigger hepcidin production and/or ferroportin downregulation, also induce the expression of HO-1. Such scenario promotes increased iron release by HO-1-mediated degradation of heme molecules along with reduced iron export by ferroportin, favoring intracellular iron accumulation. Although these mechanisms can restrict nutrient iron for extracellular pathogens, they may have the opposite effect in infections with intracellular microorganisms [4]. In addition, the pro-inflammatory signals that modulate the expression of HO-1, hepcidin, and ferroportin are also produced in several sterile inflammatory conditions, such as autoimmune diseases, ischemia-reperfusion injuries and tumors, and, therefore, in all of those conditions, intracellular iron accumulation can also occur.

As discussed in the following sections, the fluctuations in intracellular iron levels in response to the mechanisms discussed above can regulate several intracellular signaling pathways that play important roles in the modulation of inflammatory and immune responses. Therefore, the crosstalk between iron homeostasis and inflammatory/immune responses holds promise as an important target for new immunomodulatory therapies.

### 4.1. Iron Regulation of HIF1α Expression

Hypoxia inducible factors (HIFs) are alpha/beta heterodimeric transcription factors that play critical roles in the adaptive transcriptional responses to O_2_ deprivation (hypoxia). Under normoxia, the prolyl hydroxylase (PHDs) and asparaginyl hydroxylase (factor inhibiting HIF—FIH) enzymes use O_2_ as a cofactor in order to catalyze a hydroxylation reaction in the HIF-α chains that will culminate in their ubiquitination and degradation by the proteasome [104,105]. These enzymes can also use Fe^2+^ ions as cofactors, and therefore, under hypoxia or iron depletion, the hydroxylation of HIF-α subunits is inhibited and the expression of the transcription factors as a whole is stabilized, culminating in the increased expression of genes induced by them [104,106].

**Figure 2:**
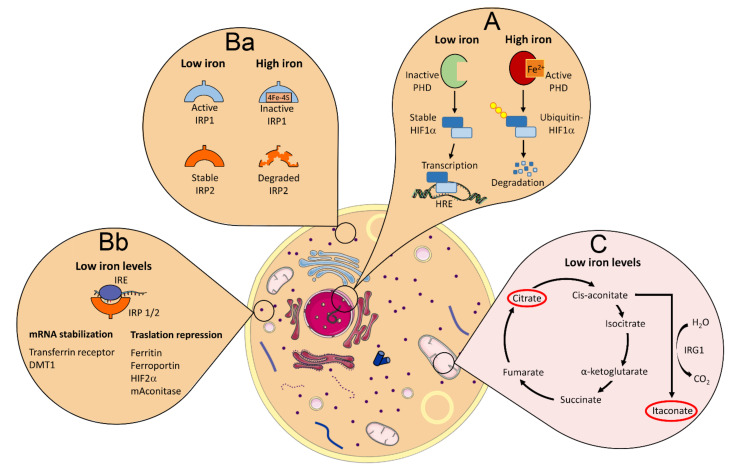
Major iron-regulated pathways involved in the modulation of inflammation and immune responses. (**A**) Prolyl hydroxylase (PHD) enzymes use iron as a cofactor to mediate the reaction that results in the ubiquitination and further degradation of HIF-1α by the proteasome in high iron concentration environment. In the presence of low iron levels, PHDs are inactivated, which results in the stabilization of HIF-1α molecules that migrate to the nucleus and promote the transcription of pro-inflammatory genes. (**Ba**) Iron regulatory proteins 1 and 2 (IRP1 and 2) are active (IRP1) or stabilized (IRP2) in low iron concentration or are inactivated (IRP1) or degraded (IRP2) in high iron concentrations. (**Bb**) Active and stable IRPs (low iron levels) bind to iron responsive element (IRE) motifs present in the untranslated region (UTR) of mRNA molecules of some proteins, which can stabilize mRNA and promote protein translation if the IRE is located at 3′ of the mRNA UTR (transferrin receptor and DMT1), or induce translation repression if the IRE is located at 5′ of the mRNA UTR (ferritin, ferroportin, HIF-2α, and mitochondrial aconitase)—high iron levels inactivate or degrade IRPs and the opposite effect is observed regarding the translation of these proteins’ mRNA molecules. (**C**) Low iron levels result in reduced translation of mitochondrial aconitase, which breaks the TCA (tricarboxylic acid) and promotes the accumulation of citrate and itaconate (highlighted with a red circle), which mediate lipid synthesis and anti-inflammatory effects, respectively. Some elements in this figure use pictures from Servier Medical Art (https://smart.servier.com, last accessed on 30 March 2022) licensed under a Creative Commons Attribution 3.0 Unported License (https://creativecommons.org/licenses/by/3.0/, last accessed on 30 March 2022).

HIF-1α and HIF-2α are the two most studied and characterized members of the HIF family of transcription factors and share high identity in their functional domains [107]. Their susceptibility to degradation under normoxia, however, is different, since HIF-2α is stabilized at higher O_2_ concentrations and for longer durations compared to HIF-1α [108]. Moreover, the modulation of HIF-1α expression in particular, is highly susceptible to changes in iron concentration, since the chelation of iron by bacterial siderophores was demonstrated to be able to induce HIF-1α stabilization and expression independently of hypoxia, while increases in iron levels induce its degradation by PHDs [109]. In fact, the use of iron chelating agents is a common positive control used in the induction of HIF1α expression (Figure 2A) [110]. HIF-1α has been demonstrated to play a role in glycolytic metabolism, apoptosis, angiogenesis, cellular stress, and inflammation, among other major biological processes [104].

Macrophages are known to adapt to hypoxic conditions, which involve the expression of genes induced by HIF-1α. Many of the alterations observed during monocyte to macrophage maturation that are induced in response to hypoxia, were shown to be driven by changes in gene expression pattern and phenotype that are dependent on the expression of HIF-1α [111,112]. In addition, aerobic glycolysis (Warburg effect) is observed in activated macrophages, in which HIF-1α expression occurs in the absence of hypoxia and plays an important role in the induction of pro-inflammatory responses [113,114,115]. In the latter situation, in particular, it is possible that fluctuations in the labile iron pool of macrophages might be involved in the regulation of HIF-1α expression through modulation of PHD activity. In fact, iron depletion of macrophages was found to mimic the effects of hypoxia, by driving HIF-1α stabilization and promoting the transcription and synthesis of IL-1β [116]. Studies have also demonstrated that treatment with the iron chelator deferoxamine induces DNA binding activity to the hypoxia-inducible factor 1 (HIF-1) consensus sequence of the iNOS gene promoter and activated the gene’s hypoxia responsive element (HRE) in murine macrophages [117]. Along with HIF-1α, iron chelators were also demonstrated to promote the activation of nuclear factor (NF)-IL6, which further induces the expression of iNOS and production of NO by macrophages [117,118].

Multiple studies have demonstrated a role for HIF-1α in the induction of pro-inflammatory cytokine production by macrophages as well as in the activation of its cellular microbicidal functions responses [111]. For instance, in *Mycobacterium tuberculosis* (Mtb)-infected mice, the blockade of HIF-1α during the early stage of infection, in which pro-inflammatory responses are critical to contain bacterial replication, results in higher susceptibility to infection. On the other hand, HIF-1α inhibition during the late stage of Mtb infection resulted in decreased bacillary loads [119], likely due to the restraining of detrimental pro-inflammatory responses, which may promote susceptibility during chronic phase of Mtb infection by inducing tissue damage. IFN-γ restricts the ability of Mtb to grow in macrophages and defects in the IFN-γ signaling pathway result in increased susceptibility to infection both in humans and mice [120]. RNA sequencing analysis demonstrated that almost half of all the genes upregulated by IFN-γ are HIF-1α-dependent genes [121].

Several studies have shown the importance of HIF-1α in metabolism, differentiation, migration, and cell survival in both hypoxia and inflammation for other immune cells as well [111]. DCs of mice with a conditional deletion of HIF-1α display a reduction in IL-22 production and CCR7 expression in response to hypoxia, which impacts the migratory capacity of these cells [122]. In neutrophils, HIF-1α promotes cell survival and phagocytosis, which might favor the control of infections with different pathogens by these cells [123]. HIF-1α has also been shown to play a role in the differentiation and function of different T-cell subsets [111]. Studies demonstrated that Th17 cells display the highest rates of HIF-1α-dependent glycolysis, while Tregs comprise the CD4^+^ T-cell subset that undergoes the lowest glycolysis levels [124]. CD8^+^ T cells lacking HIF-1α were also shown to express reduced levels of glycolytic enzymes along with decreased production of IFN-γ and TNF following TCR stimulation [125].

Therefore, HIF-1α stabilization and enhanced expression triggered by the reduction in cytosolic iron levels can play an important role in the activation of macrophage pro-inflammatory functions, and may additionally be involved in the HIF-1α-dependent activation of other immune cell subsets, such as DCs, neutrophils, and T cells.

### 4.2. Iron Regulation of IRPs/IRE Interactions

The iron-regulatory proteins (IRPs) 1 and 2 (IRP1 and IRP2) are mRNA-binding proteins that recognize and interact with non-coding sequences, known as iron responsive elements (IREs) present at the 3′ or 5′ untranslated region (UTR) of mRNA transcripts of particular genes, forming conserved RNA stem loop structures. The binding of IRPs to IREs located at 3′ regions protects the mRNA molecule from degradation and promotes its translation, while binding of IRPs to IREs at the 5′ regions blocks the translation of mRNA molecules into proteins [126,127]. Much of the cellular iron uptake, transport, storage, utilization, and release processes are controlled by the IRP/IRE system [24]. When intracellular iron is abundant, it binds to IRP1 and alters its conformation, making it incapable of binding to IREs, while high iron concentrations promote the degradation of IRP2. In situations of low iron tension, IRP1 is not bound to iron and assumes a conformation with high affinity for the IREs, while expression of IRP2 is stabilized (Figure 2Ba). Therefore, in cases of intracellular iron accumulation, mRNAs from genes that have IREs at 3′ of UTRs will be degraded, while those that have IREs at 5′ of UTRs will be translated, due to the absence of IRP binding to the IREs. When iron tension is low, IRPs are able to bind to IREs, and the opposite effect is observed [128]. The mRNAs of the iron importer proteins transferrin receptor 1 (TFR1) and DMT1 (SLC11A2) have IREs at 3′ of UTR, and therefore, their translation is increased when intracellular iron concentration is low and repressed when iron levels in the cytosol are high. On the other hand, the mRNAs for ferroportin (SLC40A1), as well as the heavy (FTH) and light (FTL) chains of ferritin have IREs at 5′ of the UTR, which results in induction of translation when intracellular iron concentration is high and repression at low iron levels (Figure 2Bb) [129,130]. The mRNA of other genes that are not involved or at least not exclusively involved in iron homeostasis also have IREs, and therefore, their translation can be regulated by the IRP/IRE system.

Aconitase is an enzyme primarily located in the mitochondria, which participates in the second step of the tricarboxylic acid (TCA) cycle, catalyzing the conversion of citrate into cis-aconitate and then isocitrate in a reversible way, while isocitrate is further decarboxylated by isocitrate dehydrogenase (IDH) into α-ketoglutarate, giving sequence to the TCA cycle in mitochondria [131]. The mRNA for the mitochondrial aconitase (Aco2) has an IRE motif at 5′ of the UTR, and therefore, its translation is induced when iron levels are high and repressed in low intracellular iron concentrations [132,133]. Cis-aconitate, the intermediate product in the conversion of citrate into isocitrate by Aco2, can be also metabolized into itaconate by the action of the enzyme cis-aconitate decarboxylase, also known as immune-responsive gene 1 (IRG1) [131,134].

Recently, it was demonstrated that iron deprivation in activated macrophages results in reduced Aco2 activity, which promotes a break in TCA cycle inducing citrate accumulation and lipid droplet formation (Figure 2C). Importantly, these iron-deprived macrophages also displayed enhanced itaconate accumulation, which resulted in decreased pro-inflammatory cytokine production [135]. Itaconate has been shown to induce important anti-inflammatory effects, such as the downregulation of Nos2, Il6, and Il12b gene expression as well as reduction of mature IL-1β and IL-18 production. Itaconate has also been shown to block IL-1β production through the inhibition of succinate dehydrogenase (SDH) activity [136]. In addition, it was found that itaconate mediates Nrf2 activation in LPS-treated mouse and human macrophages. Itaconate directly modifies proteins via alkylation of cysteine residues in Keap1, which releases Nrf2 and allows it to promote an increase in the expression of downstream genes with antioxidant and anti-inflammatory properties [137]. Therefore, the regulation of Aco2 translation by the IRP/IRE system and the resulting consequences in the modulation of itaconate levels in the cell, may be an important mechanism through which the intracellular iron levels can regulate inflammatory responses.

As mentioned previously, HIF-2α is a member of the HIF family of transcription factors. However, differently from HIF-1α, the mRNA of HIF-2α has an IRE at 5′ of the UTR and therefore, its translation is induced under high cytosolic iron concentrations [130]. HIF-2α plays an important role in iron homeostasis in living organisms by regulating the transcription of DMT1 and erythropoietin genes; the latter is critical for erythropoiesis [138]. However, such as HIF-1α, HIF-2α also plays important roles in the regulation of inflammatory responses [111].

Imtiyaz et al. have demonstrated that HIF-2α activity promotes pro-inflammatory cytokine production and mortality in a LPS-induced shock model [139]. More recently, it was observed that HIF-2α modulation of macrophage mitochondrial metabolism favors the development of pro-inflammatory responses during myocardial infarction [140]. On the opposite direction, studies have demonstrated an anti-inflammatory role for HIF-2α in infections and inflammatory diseases. During the infection with *M. marinum* in fish, the negative regulation of HIF-2α results in improved control of bacterial replication through an iNOS-dependent mechanism [141]. In fact, while HIF-1α expression is associated with M1 macrophages, which express high levels of iNOS, HIF-2α have been identified as an inducer of Arg1 expression, which is characteristic of M2 alternatively activated macrophages [142]. Additionally, Kerber et al. have demonstrated that HIF-1α and HIF-2α play opposite roles in myeloid cells during colitis. While HIF-1α was found to induce colon inflammation, HIF-2α activity was associated with the suppression of inflammatory responses. More specifically, animals with conditional deletion of HIF-2α in myeloid cells displayed increased susceptibility to colitis development, characterized by enhanced levels of neutrophils, CD4^+^ and CD8^+^ T cells as well as higher expression of IL-6 and IFN-γ compared to controls [143]. More recently, a study by Hsu et al. demonstrated that HIF-2α deletion in regulatory T cells results in a loss of their suppressive functions, which display enhanced reprogramming toward Th17 phenotype. Additionally, the authors demonstrated that mice carrying HIF-2α deletion in Tregs are more resistant to tumors [144].

The studies cited above clearly demonstrate that the role played by HIF-2α in the modulation of immunity and inflammation varies according to the cells and tissues in which the reaction takes place. Therefore, the effects of iron-induced regulation of HIF-2α expression might likely play different roles in the modulation of inflammation and immunity depending on the target organ.

### 4.3. Iron Induction of ROS Generation by Fenton Reaction

Macrophages produce inflammation-related proteins, such as myeloperoxidase, NADPH oxidase, indoleamine 2,3-dioxygenase, nitric oxide synthases, or lipoxygenases, all of which contain iron [145]. Moreover, iron induces the generation of ROS by the Fenton reaction and is also involved in the production of such radicals by the phagocyte oxidase. NOX2, a NOX family member that is part of the phagocyte oxidase system, is a transmembrane hemoprotein that uses heme iron to transport electrons across membranes to catalyze the generation of superoxide (**•**O_2_), via the following reaction: O_2_ + **•**O_2_. When NOX2 accumulates in macrophages, **•**O_2_ can give rise to other ROS, such as hydrogen peroxide (H_2_O_2_), which reacts with iron to generate hydroxide ions (OH^−^) and hydroxyl radicals (**•**OH), leading to the production of hydrogen peroxide radicals (HOO**•**). This latter step occurs via two iron-catalyzed reactions, first Fe^2+^ + H_2_O_2_ → Fe^3+^ + HO^−^ + **•**OH and second (Fe^3+^ + H_2_O_2_ → Fe^2+^ + HOO**•** + H^+^). These ROS play a critical role in the destruction of pathogens in phagolysosomes but also support other macrophage functions, such as disassembling of dying cells internalized by phagocytosis [8,146]. Accordingly, it was demonstrated that Fe chelation dramatically exacerbates murine infection with *S. typhimurium* via inhibition of the host phagocyte oxidase-dependent respiratory burst and the production of nitrogen radical catalyzed by the inducible nitric oxide synthase [147].

HO-1 has been demonstrated to play an important role in cancer. The antioxidant properties of HO-1 are involved in the protection of cancer cells against oxidative stress, which favors tumor survival and progression [148,149]. The enzyme also promotes angiogenesis, which favors tumor invasiveness and metastasis, while expression of HO-1 in tumor-associated macrophages (TAM) was found to impair immune responses and favor tumor progression as well [150,151,152,153,154,155,156,157]. However, HO-1 expression may also have an opposite effect in cancer, in particular considering its role in the release of iron following heme degradation. Intracellular accumulation of Fe^2+^ and subsequent Fenton reaction-induced ROS generation, along with decreased expression of the enzyme glutathione peroxidase 4 (GPX4), result in membrane lipid peroxidation and necrotic cell death by ferroptosis [158,159]. Excessive expression of HO-1 has been shown to promote intracellular iron accumulation and oxidative stress [160]. Chang et al. have demonstrated that the toxicity of the compound BAY11-7085 against tumor cells occurs through the induction of ferroptotic cell death and that HO-1 plays a major role in this effect, by promoting intracellular iron accumulation [161].

The ferrous iron and Fenton reaction-induced hydroxyl radical formation, as mentioned above also drives oxidative damage and contributes to cellular injuries. To maintain homeostasis in healthy cells, the Nrf2 transcription factor plays a critical role in the regulation of iron-mediated oxidative damage through the induction of several antioxidant cytoprotective mechanisms, and among them, the expression of ferroportin and ferritin, which are directly related to the control of iron accumulation-induced detrimental effects [162,163].

### 4.4. Iron and Polarization of M1 and M2 Macrophages

M1 macrophage polarization is induced in response to Th1 cytokines, such as TNF and IFN-γ, or by bacterial LPS recognition. These macrophages produce majorly pro-inflammatory cytokines, such as TNF, IL-1α, IL-1β, IL-6, IL-12, IL-23 and low levels of IL-10. M1 macrophages display potent anti-microbial activity mainly through the generation of NADPH-oxidase-dependent ROS and iNOS-induced NO production [164].

M2 macrophages display an anti-inflammatory profile and are polarized in response to Th2 and suppressor cytokines, such as IL-4, IL-13, and IL-10. IL-33 is another cytokine involved in M2 polarization, through the amplification of IL-13-induced effects. M2 macrophages are characterized by the high expression of arginase-1 (Arg-1) and production of both IL-10 and TGF-β, while the production of pro-inflammatory cytokines is very low or absent. M2 macrophages play an important role in the scavenging of cellular debris and apoptotic cells, as well in the promotion of tissue repair and wound healing, besides displaying pro-angiogenic and pro-fibrotic properties [164].

M1 and M2 macrophages have been characterized regarding their iron status and IRP/IRE binding activity. RNA binding activity of IRPs was found to be low in M1 macrophages, which present intracellular iron accumulation, while M2 macrophages were found to display high IRP/IRE binding activity, which was associated with lower intracellular iron levels [165]. The molecular signature of M1 macrophages, in which ferroportin expression is reduced and ferritin H is upregulated, favors iron sequestration and this effect is observed in the reticuloendothelial system in several inflammatory disorders. On the other hand, M2 macrophages have an expression profile characterized by upregulation of ferroportin and HO-1 expression along with the downregulation of ferritin H, which correlates with enhanced release of iron, and therefore, reduced retention of the metal in the intracellular compartment [166]. Iron overload, induced by treatment with ferric citrate, has been shown to induce M1 polarization in RAW 264.7 macrophages, which was associated with the induction of ROS production in iron-treated cells [167]. In an experimental model of chronic venous ulcers, the induction of iron overload in macrophages was shown to potently induce M1 polarization, which was characterized by elevated TNF production and to promote poor wound healing properties in these cells. In addition, the pro-inflammatory activity of these macrophages promoted DNA damage and senescence of skin-resident fibroblasts [168]. These results are in agreement with the characterization of M1 macrophages as cells that accumulate intracellular iron [166], and in addition, indicate that the cytosolic iron levels may play a role in driving such M1 polarization program.

However, a study by Agoro et al. demonstrated that, contrarily to the studies cited above, the expression of Arg1 and IL-10 along with a series of genes associated with M2 polarized macrophages, such as Ym1, IL-10, and Stat6, were all upregulated in mice fed with a diet containing high levels of iron. On the other hand, mice that were fed a diet poor in iron displayed increased production of pro-inflammatory cytokines and expression of M1 macrophage markers [169]. Along these same lines, studies employing experimental in vitro and in vivo models of *Salmonella* infection have demonstrated that a major mechanism used by macrophages to restrict infection is to enhance the expression of ferroportin, therefore decreasing the intracellular iron pool, which also enhances the levels of iNOS expression and NO production in response to the reduction of intracellular iron concentration [170,171,172].

The studies discussed above demonstrate that although some pattern is observed in M1 and M2 macrophages regarding the expression of molecules linked to the modulation of iron homeostasis, it is hard to draw a definite conclusion on whether different iron levels can be is associated with M1 or M2 macrophage phenotypes. Regardless, these studies clearly demonstrate that the levels of cytosolic iron can play important roles in the regulation of pro- and anti-inflammatory programs of macrophages.

## 5. Conclusions

HO-1 plays a key role in maintaining cellular homeostasis, particularly through its anti-inflammatory and antioxidant properties, which were proven to display several cytoprotective functions throughout the organism [1,149]. However, HO-1 activity also results in the release of pro-oxidant ferrous iron (Fe^2+^). In fact, the recycling of iron from heme molecules in macrophages by the action of HO-1 is the major mechanism used by the organism to acquire the metal. The coordinated action of HO-1 and the hepcidin/ferroportin axis controls the release of iron to the serum and/or its retention inside cells to efficiently maintain optimal systemic iron levels [97].

As discussed in this review, alterations in iron homeostasis can have profound impacts on the regulation of inflammation and immune responses. In particular, the changes in macrophage intracellular iron levels resulting from modulation of HO-1 expression and activity as well as from the transcriptionally or hepcidin production-induced regulation of ferroportin expression, can impact the activation of microbicidal effector functions as well as cytokine production by these cells. Given the important role played by macrophages in the pathogenesis of several autoimmune and auto-inflammatory disorders, as well as in the host response to infectious diseases with different pathogens [145,173], the iron metabolism of macrophages represents a potential target for novel immunomodulatory therapeutic strategies in these areas. In recent years, several advances have been achieved in the identification of novel inhibitors and inducers of HO-1 activity as well as in modulators of the hepcidin/ferroportin axis [174,175,176]. Besides their use in therapies to treat disorders in systemic iron homeostasis, in particular different forms of anemia, this review highlights additional potential applications for those drugs, such as in host-directed therapies to treat infections and immunomodulatory interventions in autoimmune and auto-inflammatory diseases as well as in cancer.
